# A Case Report of Acute-on-Chronic Methemoglobinemia

**DOI:** 10.5811/cpcem.31025

**Published:** 2025-01-01

**Authors:** Gabriel Lathrop, Rodney Fullmer

**Affiliations:** Endeavor Health Swedish Hospital, Department of Graduate Medical Education, Chicago, Illinois

**Keywords:** acquired methemoglobinemia, congenital methemoglobinemia, cytochrome b5 reductase deficiency, toxicology emergency

## Abstract

**Introduction:**

Methemoglobinemia is a rare hematologic disorder of hemoglobin, in which iron contained within the heme moiety becomes oxidized from ferrous iron to ferric iron at a concentration greater than 1% in the blood. This biochemical change reduces binding affinity for oxygen, leading to impaired oxygen deposition in tissues and subsequent hypoxia and hypoxemia. The etiology of methemoglobinemia is often acquired from exposure to oxidizing agents, commonly antibiotics such as dapsone or local anesthetics such as benzocaine. A rare cause results from congenital deficiency of cytochrome b5 reductase, a nicotinamide adenine dinucleotide dependent enzyme within red blood cells that donates electrons to reduce ferric to ferrous iron.

**Case Report:**

A 22-year-old previously healthy female was referred to the emergency department (ED) by her dentist one week after a dental procedure where she was noted to have low oxygen saturation and dark blood upon reported exposure to benzocaine. Upon arrival to the ED one week after exposure, her vitals were notable for oxygen saturation of 89% on room air. She was placed on 6 liters supplemental nasal cannula oxygen with subsequent improvement of oxygen saturation to 92%. Her exam was concerning with pale appearance, perioral cyanosis, and dusky fingertips. Her laboratory studies were most notable for serum methemoglobin level critically elevated to 31.6% one week after exposure, and she received 1 milligram per kilogram methylene blue in the ED with subsequent reduction of methemoglobin to 0.7%. The patient’s inpatient workup revealed a congenital deficiency in cytochrome b5 reductase.

**Conclusion:**

Methemoglobinemia arises when there is a mismatch between the formation of oxidized ferric iron and the subsequent reduction to ferrous iron. Classically, methemoglobinemia is an acquired pathologic process from acute exposure to any number of oxidative stressors; in rare cases, methemoglobinemia is caused by congenital deficiency in red blood cell-reducing enzymes. We report a case of an acquired methemoglobinemia with prolonged methemoglobinemia in a patient with undiagnosed congenital methemoglobinemia from cytochrome b5 reductase deficiency.

## INTRODUCTION

Methemoglobinemia is a rare hematologic disorder of hemoglobin in which levels of methemoglobin (MetHb) are greater than 1% concentration in the blood.^1^ This occurs when iron within the heme moiety becomes oxidized, from Fe^2+^ (ferrous iron) to Fe^3+^ (ferric iron), thereby decreasing hemoglobin’s affinity for oxygen. Red blood cells inherently possess the ability to reduce ferric iron in MetHb to the ferrous iron found in native hemoglobin. Cytochrome b5 reductase is the enzyme responsible for restoring ferric iron and the heme’s natural oxygen-binding affinity.^2^,^3^ Methemoglobinemia develops when the rate of oxidation exceeds the rate of reduction.^3^,^4^

The majority of cases of methemoglobinemia are acquired, that is, they are due to an environmental exposure to oxidizing compounds. The list of oxidizing compounds includes many iatrogenic drugs, such as topical anesthetics (tetracaine, benzocaine), urinary analgesic (phenazopyridine), antibiotics (dapsone, nitrofurantoin), and antimalarials (chloroquine, hydroxychloroquine).^3^,^4^ In rare cases, methemoglobinemia can result from recessively inherited cytochrome b5 reductase deficiency, rendering the body unable to reduce MetHb to native hemoglobin after exposure to environmental oxidative stressors.^3^–^7^ This ultimately leads to a leftward shift of the oxygen-hemoglobin dissociation curve, decreased oxygen deposition in tissues, and subsequent hypoxemia.^2^

Clinically, a patient with methemoglobinemia will be hypoxic and cyanotic. The patient may present with a variety of cyanotic signs reflective of hypoxemia, such as ashen skin appearance and blue-tinged mucosal surfaces. Objectively, they will develop compensatory vital sign changes with tachycardia and tachypnea.^2^–^7^ They will have a low peripheral oxygen saturation, when compared to oxygen saturation by arterial blood gas, that does not respond to 100% fraction of inspired oxygen supplemental oxygen.^4^,^7^ The diagnosis is confirmed with a blood MetHb level greater than 1%.

Treatment for most cases of methemoglobinemia is focused on supportive care, with 1–2 milligrams per kilogram (mg/kg) methylene blue indicated for patients with critically elevated concentration of MetHb (usually greater than 30% in asymptomatic individuals, or greater than 20% in symptomatic patients, or for patients at high risk for decompensation if left untreated).^1^,^3^,^7^,^9^ Methylene blue is contraindicated in patients with glucose-6-phosphate dehydrogenase deficiency due to risk of hemolytic anemia and, thus, a thorough family history is important when interviewing a possible candidate.^1^ Alternative treatment options for patients in which first-line therapy is not indicated, or is otherwise ineffective consist of hyperbaric oxygen, exchange transfusion, and supplementation with ascorbic acid (vitamin C).^1^,^8^,^9^ Intravenous dextrose can be used as an adjunct therapy that is administered to generate nicotinamide-adenine dinucleotide (via glycolysis) and nicotinamide adenine dinucleotide phosphate (NADPH) (via pentose phosphate pathway).^10^ These electron donors serve as important cofactors for reduction of MetHb via the cytochrome b5 reductase, and NADPH-MetHb reductase, respectively.^1^,^10^

## CASE REPORT

A previously healthy 22-year-old female presented to the emergency department (ED) by referral from her dentist one week after wisdom tooth extraction for chief complaint of “abnormal vital sign.” She was informed by her dentist that her oxygen saturation was low during her procedure and that her blood had a very dark appearance. The patient was unsure of what type(s) of local anesthesia was used during the procedure. Topical benzocaine gel was applied to the extracted tooth socket postoperatively. Upon arrival to the ED, the patient’s vital signs showed a blood pressure 127/74 millimeters of mercury, heart rate 77 beats per minute, respiratory rate 20 breaths per minute, temperature 36.9º Celsius, and oxygen saturation of 89% on room air, which improved marginally to 92% with six liters supplemental nasal cannula oxygen. Physical exam was notable for pale-appearing female without respiratory distress who displayed cyanosis at the lips and distal extremities.

**CPC-EM Capsule**

What do we already know about this clinical entity?

*Methemoglobinemia is a hematologic state in which the rate of iron oxidation exceeds iron reduction, leading to decreased oxygen-binding affinity*.

What makes this presentation of disease reportable?

*The patient’s undiagnosed congenital methemoglobinemia did not permit natural reduction to hemoglobin*.

What is the major learning point?

*Patients with congenital methemoglobinemia may develop methemoglobinemia after exposure to non-toxic levels of reducing agents and present with a prolonged methemoglobin state*.

How might this improve emergency medicine practice?

*Congenital methemoglobinemia patients may have chronically elevated levels and become acutely symptomatic upon exposure to reducing agents*.

On review, the patient endorsed a long history of dyspnea on exertion (DOE), poor exercise tolerance, discolored blue lips, and dusky fingertips that were worsened by exposure to cold temperature. She had previously presented to the same ED with similar symptoms of cyanosis and DOE with pleuritic chest pain approximately three years prior. Documentation from that visit showed she underwent a negative cardiopulmonary workup, including D-dimer. At that time, she initially presented with oxygen saturation of 91% and was started on supplemental oxygen and given albuterol-ipratropium nebulizer treatment. The oxygen saturation sensor was then moved to the patient’s ear, which resulted in an improvement to 98%, and the patient was discharged. There was a family history of monoclonal gammopathy of undetermined significance in her grandmother, but otherwise she denied any known family history of hematologic or rheumatic diseases. Her medication list consisted of fluoxetine, tramadol, acetaminophen-codeine No. 3, and amoxicillin. She was coronavirus disease 2019-vaccinated and denied any recent illness or sick contacts.

Laboratory workup was remarkable for critically elevated MetHb level of 31.6% (reference range: 0.4–1.5%). Arterial blood gas showed oxygen saturation of 67.3% (94–97%). The patient was given 1 mg/kg methylene blue, with subsequent reduction of MetHb to 0.7%. She was admitted to the hospital for observation and further hematologic workup, which revealed reduction in activity of cytochrome b5 reductase to 2.1 units per gram (U/g) hemoglobin (Hb) (7.8–13.1 U/g Hb). Iron studies notable for an iron level of 34 micrograms per deciliter (mg/dL) (42–135 mg/dL), iron saturation of 10.8% (15–50%), and ferritin of 7.0 nanograms per milliliter (ng/mL) (10–291 ng/mL). Glucose-6-phosphate dehydrogenase enzyme activity was normal. The patient was started on supplemental 1,000 mg ascorbic acid three times daily and discharged from the hospital in stable condition. Outpatient follow-up showed resolution of her microcytic anemia with normalization of iron level and iron saturation. The patient continues to be on vitamin C maintenance therapy and receives methylene blue infusions monthly. She had one subsequent hospital admission due to maintenance therapy noncompliance and was admitted for methylene blue infusion with MetHb level of 26.8%.

## DISCUSSION

Methemoglobinemia is an extremely rare and potentially life-threatening emergency if unrecognized. It is widely accepted that most cases arise from exposure to oxidizing agents such as dapsone, hydroxychloroquine, benzocaine, and nitrate/nitrite substances.^1^,^4^,^6^,^7^ Healthy individuals with intact cytochrome b5 reductase activity can reduce MetHb upon its formation. Methemoglobinemia may arise when the formation of MetHb exceeds the enzymatic rate of reduction, building up to serum levels greater than 1.5%.^1^ Without reduction ability, methemoglobinemia can arise from otherwise innocuous exposure to an oxidizing compound and stay elevated until there is an appropriate medical intervention, or death—whichever comes first.

We report the first documented case of a patient with an undiagnosed, congenital methemoglobinemia and superimposed acquired methemoglobinemia who presented with prolonged methemoglobinemia one week following benzocaine exposure. The exposure one week prior triggered hemoglobin iron oxidation that could not be reduced to the ferrous state because of a congenital deficiency of cytochrome b5 reductase. With her rare enzymatic deficiency unknown, this patient maintained critically elevated MetHb levels for a week prior to her presentation in the ED.

## CONCLUSION

In the majority of acute methemoglobinemia cases, there will be history of an oxidative exposure from the prior 24–48 hours. This can be iatrogenic—from a prescribed antibiotic or antimalarial, over-the-counter pharmaceuticals such as phenazopyridine and benzocaine gel—or it may be occupational exposure from aniline dye used in varnish, or nitrate/nitrites found in the meat-packing industry. The constellation of chocolate-brown blood, refractory hypoxia, and saturation-cyanosis gap are clinical clues that may assist emergency physicians in early recognition of methemoglobinemia. The details of this unique case emphasize that populations with congenital methemoglobinemia may sustain prolonged methemoglobin levels for several days, if not weeks, after an inciting event.

## References

[1] Rehman HU (2001). Methemoglobinemia. West J Med.

[2] Wright RO, Lewander WJ, Woolf AD (1999). Methemoglobinemia: etiology, pharmacology, and clinical management. Ann Emerg Med.

[3] Tintinalli JE, Stapczynski J, Ma O (2020). Tintinalli’s Emergency Medicine: A Comprehensive Study Guide.

[4] Skold A, Cosco DL, Klein R (2011). Methemoglobinemia: pathogenesis, diagnosis, and management. South Med J.

[5] Hegesh E, Hegesh J, Kaftory A (1986). Congenital methemoglobinemia with a deficiency of cytochrome b5. N Engl J Med.

[6] Mansouri A, Lurie AA (1993). Concise review: methemoglobinemia. Am J Hematol.

[7] Haymond S, Cariappa R, Eby CS (2005). Laboratory assessment of oxygenation in methemoglobinemia. Clin Chem.

[8] Cortazzo JA, Lichtman AD (2014). Methemoglobinemia: a review and recommendations for management. J Cardiothorac Vasc Anesth.

[9] Goldfrank LR, Flomenbaum NE, Lewin NA (1998). Price D. Methemoglobinemia. Goldfrank’s Toxicologic Emergencies.

[10] Zuschlag ZD, Warren MW, Schultz KS (2018). Serotonin toxicity and urinary analgesics: a case report and systematic literature review of methylene blue-induced serotonin syndrome. Psychosomatics.

